# Modulation, Bioinformatic Screening, and Assessment of Small Molecular Peptides Targeting the Vascular Endothelial Growth Factor Receptor

**DOI:** 10.1007/s12013-014-0151-x

**Published:** 2014-07-29

**Authors:** Shibin Feng, Lingyun Zou, Qingshan Ni, Xiang Zhang, Qianwei Li, Lei Zheng, Laiping Xie, Hongmin Li, Dingde Huang

**Affiliations:** 1 Department of Nuclear Medicine, Southwest Hospital, Third Military Medical University, 30 Gaotanyan Street, Shapingba District, Chongqing, 400038 China; 2 Department of Microbiology, College of Basic Medical Sciences, Third Military Medical University, 30 Gaotanyan Street, Shapingba District, Chongqing, 400038 China

**Keywords:** Bioinformatics, Molecular probe, Tumor, VEGF/VEGFR

## Abstract

Vascular endothelial growth factor (VEGF) and VEGF receptor (VEGFR) are important factors in tumor growth and metastasis. Molecular probes or drugs designed to target VEGF/VEGFR interactions are crucial in tumor molecular imaging and targeted therapy. Bioinformatic methods enable molecular design based on the structure of bio-macromolecules and their interactions. This study was aimed to identify tumor-targeting small-molecule peptides with high affinity for VEGFR using bioinformatics screening. The VEGFR extracellular immunoglobulin-like modules Ig1–Ig3 were used as the target to systematically alter the primary peptide sequence of VEGF_125–136_. Molecular docking and surface functional group interaction methods were combined in an in silico screen for polypeptides, which in theory, would have higher affinities for VEGFR. In vitro receptor competition binding assays were used to assess the affinity of the putative VEGFR-binding polypeptides. Rhodamine-conjugated peptides were used to label and visualize peptide-binding sites on A549 cells. Using bioinformatic screening, we identified 20 polypeptides with potentially higher affinity for VEGFR. The polypeptides were capable of inhibiting the binding of ^125^I-VEGF to VEGFR in a dose-dependent manner. The IC_50_ values of QKRKRKKSRKKH and RKRKRKKSRYIVLS (80 and 185 nmol/L, respectively) were significantly lower than that of VEGF_125–136_ (464 nmol/L); thus, the affinity of these peptides for VEGFR was 6- and 2.5-fold higher, respectively, than that of VEGF_125–136_. Rhodamine labeling of A549 cells revealed peptide binding mainly on the plasma membrane and in the cytoplasm. Bioinformatic approaches hold promise for the development of molecular imaging probes. Using this approach, we designed two peptides that showed higher affinity toward VEGFR. These polypeptides may be used as molecular probes or drugs targeting VEGFR, which can be utilized in molecular imaging and targeted therapy of certain tumors.

## Introduction

In tumor diagnosis and therapy, the discovery of disease-relevant molecular targets and the construction of molecular probes or targeted drugs with high specificity for these targets are crucial [1, 2]^.^. Certain molecules, including vascular endothelial growth factor receptor (VEGFR), integrin α_v_β_3_ [3, 4], somatostatin receptor [5], vasoactive intestinal peptide receptor [6], matrix metalloproteinases [7], E-selectin [8], and CD105 [9], are expressed at higher levels in tumor cells and in newly formed vascular endothelial cells. Thus, these molecules are often used as targets in tumor-targeted radionuclide imaging or therapy [10]. Among these molecules, vascular endothelial growth factor (VEGF) is the principal factor mediating tumor growth, angiogenesis, and metastasis. Molecular probes or targeted drugs based on VEGF or VEGFR can be widely applied in tumor-targeted molecular imaging or therapy [11–17].

VEGF_125–136_ is a 12-amino acid peptide (QKRKRKKSRYKS) encoded by the VEGF-A gene (exon 6). VEGF_125–136_ binds specifically to VEGFR, but does not activate its signal pathway [18, 19]. Our previous study demonstrated that VEGF_125–136_ exhibits good tumor-targeting properties, and could be used as a highly specific agent for tumor radionuclide imaging and therapy [20]. However, the previous in vitro study of VEGF_125–136_ suggested that it has a relatively weak capacity for tumor growth inhibition and exhibits a relatively short retention time in tumor tissues. Molecular probes and tumor-targeting drugs need to possess high binding affinity as well as a long half-life in the tumor tissues; hence, we aimed to modify VEGF_125–136_ to improve its affinity for VEGFR.

The current study utilized a combination of bioinformatics and in vitro experimentation to improve VEGF_125–136_. Two polypeptides with higher VEGFR-binding affinity were selected from the peptide candidates, which may be used as molecular probes or targeted drugs.

## Materials and Methods

### Analysis of Molecular Docking and Surface Functional Group Interaction

#### Experimental Platform

**Fig. 1:**
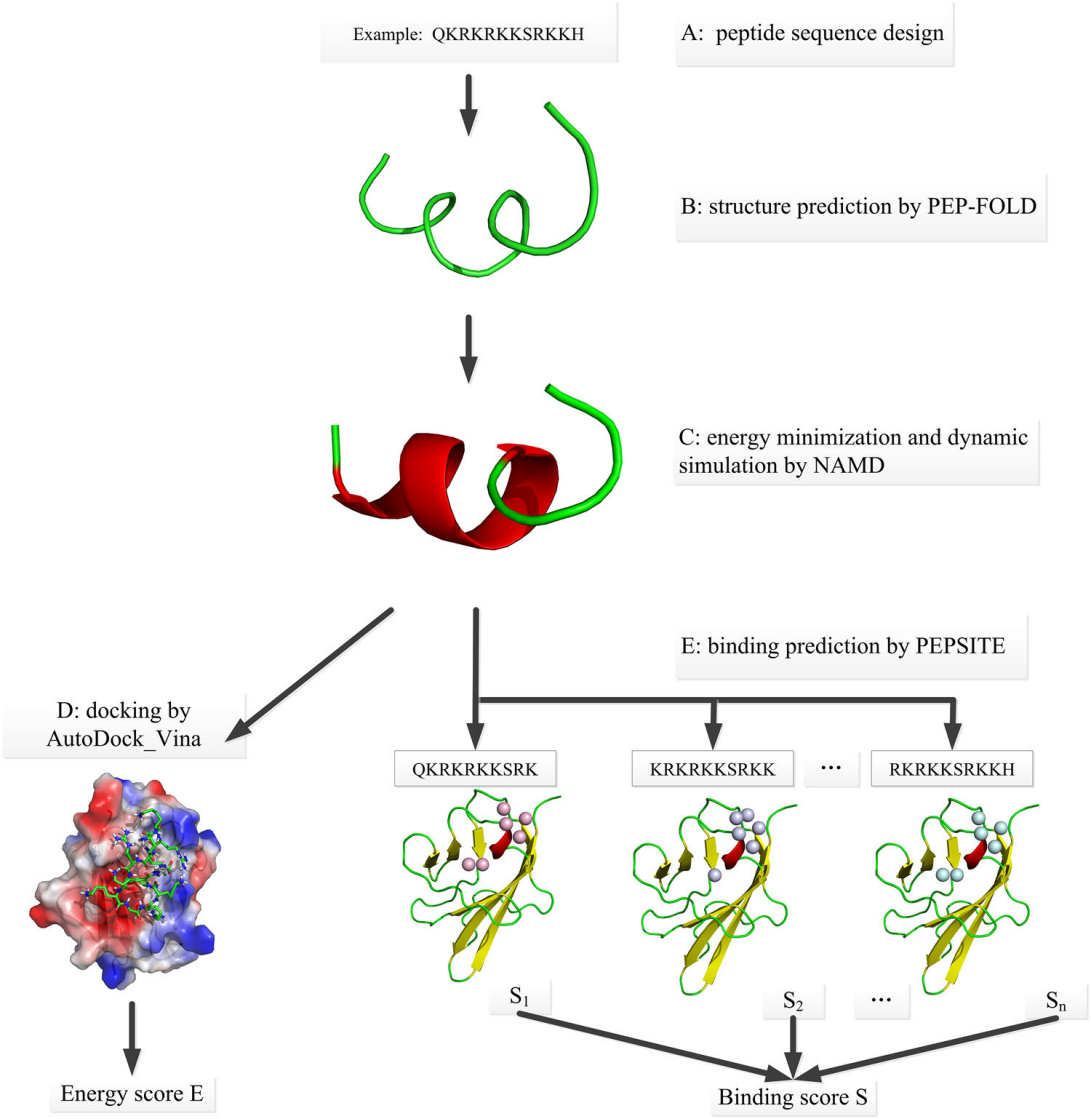
Scheme of bioinformatic screen for peptides with elevated VEGFR-binding affinity. **a** Peptide sequences are generated by manual design (here, the peptide “QKRKRKKSRKKH” is used as an example); **b** the initial structure of each peptide is predicted using PEP-FOLD software; **c** a stable peptide structure is generated by molecular dynamics simulation using NAMD (5000 steps of energy minimization and 500-ps dynamics simulation); **d** a docking procedure to VEGFR is performed for each peptide using AutoDock Vina software and scores assigned according to the output energy (here, the docking site between VEGR and “QKRKRKKSRKKH” with minimal energy output is shown); **e** sub-sequences cut from the peptide with a sliding-window method used to predict binding specificity using the PEPSITE algorithm, are scored using Eq. (1), and the total binding score *S* is calculated using Eq. (2) (the residues of the sub-sequences from “QKRKRKKSRKKH” are shown as spheres binding to the Ig1–Ig3 domains of VEGR)

The analyses were performed using the high-performance computing system at the Bioinformatics Center of Third Military Medical University (Chongqing, China), which is based on the open 64-bit Linux parallel computing system Rocks Cluster 5.3 (http://www.rocksclusters.org/). The system consists of 26 dual quad-core servers (208 computing cores) and is capable of ≈2 TFLOPS. The scheme used for bioinformatic screening of peptides with high VEGFR-binding affinity is shown in Fig. 1. AutoDock-Vina 1.1 software was used for molecular docking analysis [21], while PyMol and the PEPSITE program [22] were used for surface functional group interaction analysis and prediction.

#### Molecular Docking-Based Screening for Polypeptides with Enhanced VEGFR Affinity

Based on the “lock-and-key” principle of the interaction between ligands and receptors, molecular docking methods simulate the interaction between a small-molecule ligand and a macromolecular receptor. The extracellular immunoglobulin-like modules Ig1–Ig3 of VEGFR reside in the ligand-binding domain and appear to form a rigid structure, whereas the ligand was observed to have a flexible structure. In our docking computation, we assumed that altered polypeptides based on VEGF_125–136_ would interact with VEGFR in the same region. The binding energy of VEGFR and the polypeptides was calculated using AutoDock-Vina software, where lower binding energy indicates higher affinity interactions.

The initial approximate structure of VEGF_125–136_ was predicted by PEP-FOLD (http://bioserv.rpbs.univ-paris-diderot.fr/PEP-FOLD/), and NAMD (version 2.7, http://www.ks.uiuc.edu/Research/namd/) was employed in the molecular dynamics simulation to obtain a refined structure. During molecular dynamics simulations, all peptide atoms were surrounded with a cubic water box of simple point-charge water molecules that extended 10 Å from the protein, and periodic boundary conditions were applied in all directions. The systems were neutralized with Na^+^ and Cl^−^ counter ions replacing the water molecules, and a 5,000 step energy minimization was performed, followed by a 500-ps production molecular dynamics simulation with a time-step of 2 fs at constant pressure (1 atm) and temperature (300 K).

First, the binding energy of VEGF_125–136_ to VEGFR was calculated and used as the reference free energy. Then, leaving the core motif RKRKKSR of VEGF_125–136_ unaltered, we sequentially mutated the amino acids at the 1st, 2nd, 10th, 11th, and 12th positions of the sequence. The total number of mutations was 20 × 20 × 20 × 20 × 20 = 3.2 × 10^6^. A hill-climbing algorithm was adopted to reduce the search range in calculating the molecular docking configuration and free binding energy between each mutated polypeptide and the receptor, and finally to screen for peptide sequences with lower binding energy.

#### Surface Functional Group Interaction Analysis Based on VEGF_125–136_

**Fig. 2:**
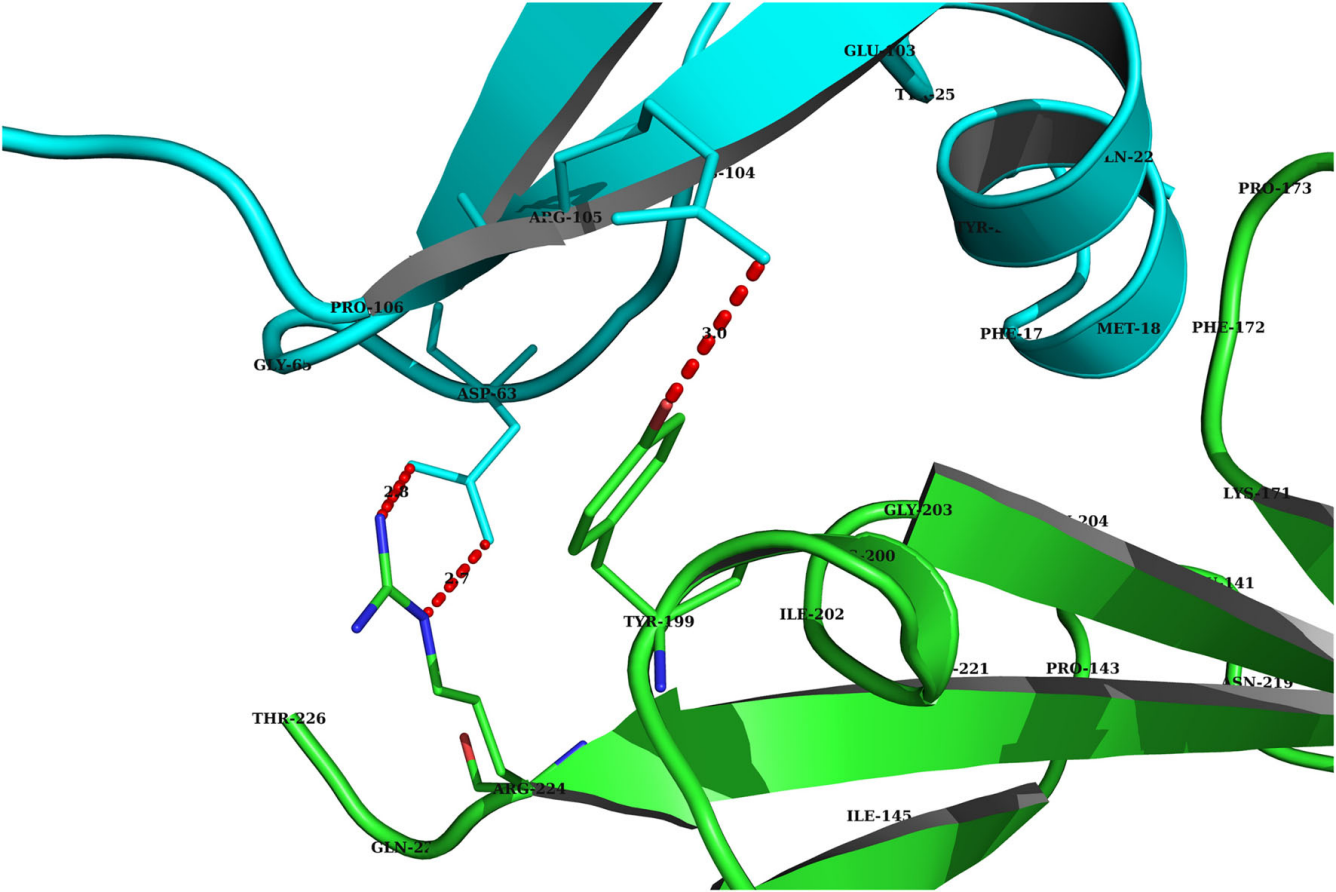
Binding of VEGF and VEGFR as analyzed with PyMol. This interaction view was created using the dimer complex structure of VEGF and VEGFR (PDB id: 1FLT). The molecular structure is shown as a cartoon with *green* indicating the extracellular domain of VEGFR (chain X) and cyan indicating VEGF (chain W). All of the residues within a distance of <5 Å between VEGF and the receptor have been labeled. All possible hydrogen bonds with a distance of <3.2 Å between VEGF and the receptor are shown as *red dotted lines*, where their lengths are calculated and labeled (Color figure online)

**Fig. 3:**
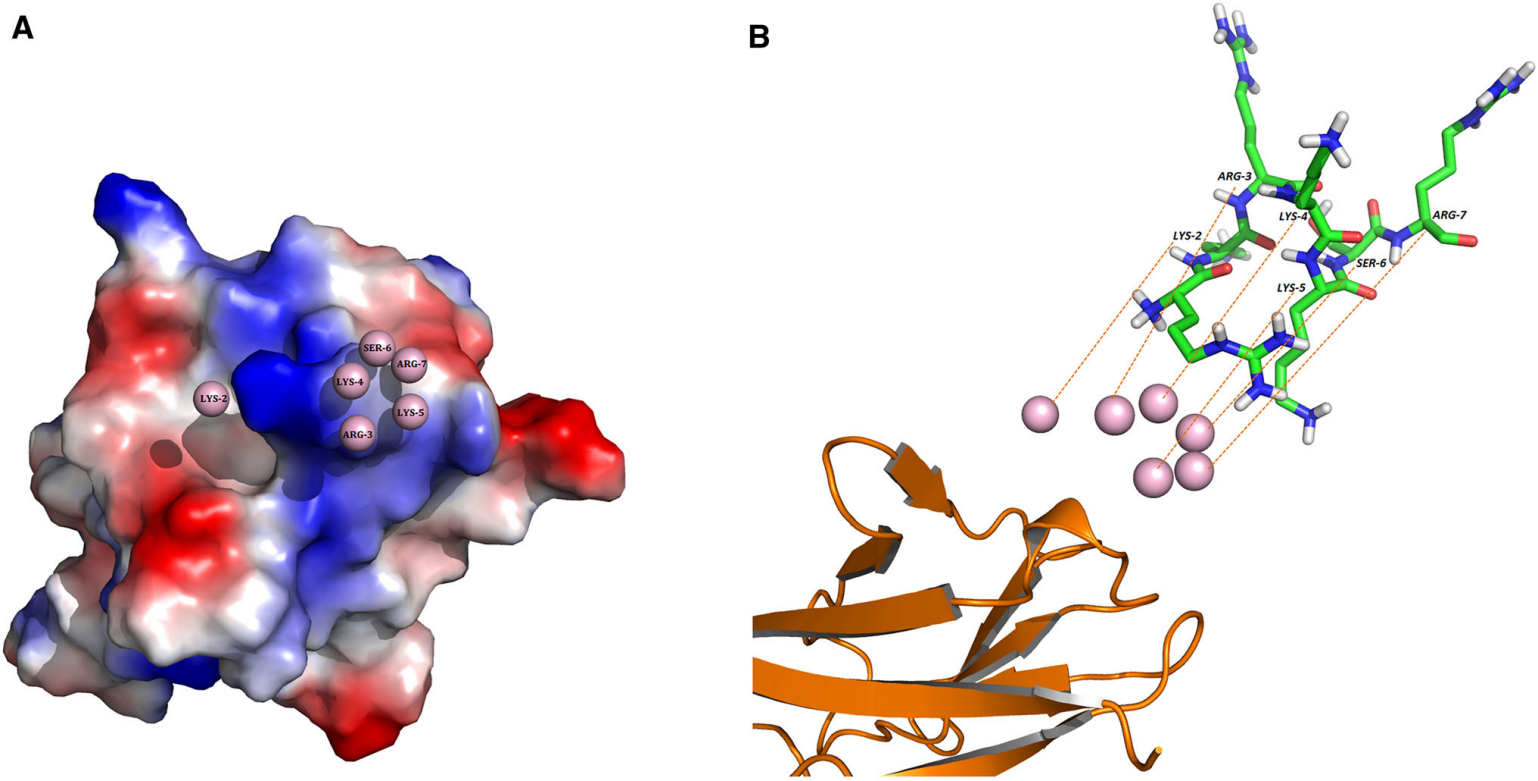
Predicted binding sites of “RKRRKSR” core motif on the surface of VEGFR. The binding sites of “RKRKKSR” core motif on the VEGFR surface as predicted by the PEPSITE program. The six balls indicate the predicted locations of six residues from “RKRRKSR”. **a** distribution of surface polar (*white*), acidic (*red*), basic (*blue*), and non-polar (*white*) groups in the extracellular domains of VEGFR. **b** the PEPSITE-binding pattern of the core motif and the extracellular domains of VEGFR (*orange*: extracellular Ig1–Ig3 domains of VEGFR, namely, chain X of VEGFR from the complex crystal structure of VEGF and VEGFR). The structure of the core motif predicted by PEP-FOLD and simulated by NAMD is shown as sticks in the figure. Each binding residue is linked with the same residue in the structure using *dotted lines*. The image shows that residues in the core peptide matched well with the predicted binding sites (Color figure online)

Studies have shown that the principle underlying the bonding between VEGF and VEGFR is based on ionic bonds and hydrophobic effects. Using PyMol, we analyzed the detailed interaction between VEGF and VEGFR (shown in Fig. 2). The surface of the peptide consists of numerous basic groups, resulting in strong interactions with the acidic groups in the extracellular domain of VEGFR. We also predicted the binding sites of “RKRKKSR” core motif on the extracellular Ig1–Ig3 domains of VEGFR using PEPSITE program. The results show that six residues of the core motif are apt to bind near to the Ig2 domain and matched well with the structure of the motif (shown in Fig. 3b), which enables the PEPSITE algorithm to accurately predict the binding sites of peptides on protein surfaces. PyMol analysis of the distribution of the extracellular Ig1–Ig3 domain surface groups revealed that hydrophobic pockets existed in proximity to multiple acidic groups (Fig. 3a). Therefore, we postulated that the fundamental approach in modulating surface group interaction should involve adding more basic hydrophilic amino acids to the VEGF_125–136_ sequence, thereby increasing the interactions with acidic groups on the surface of VEGFR. Moreover, we added hydrophobic fragments to the termini of the polypeptide to achieve a stronger interaction with the hydrophobic pockets surrounding the acidic groups. Subsequently, the bonding specificity between the newly designed peptide sequences and the receptor surface was predicting using PEPSITE, and peptide sequences that bound to VEGFR with low *p* values were obtained. Moreover, we developed a scoring scheme based on the PEPSITE prediction results. For the PEPSITE prediction, a window (length *L* ≤ 10 residues) was slid on the peptide sequence, and several sub-sequences with *L* residues were obtained (peptide length is limited to ten residues in PEPSITE). Each sub-sequence was used to predict binding specificity. The top ten results were employed to calculate binding scores (*S*) for the sub-sequences, which were determined using the following equation:

1 $$ S = \sum\limits_{i = 1}^{n} {(\frac{1}{{p_{i} }}} ), $$

where *n* is the number of all top binding sites (*n* is 10 in this study); *p*_*i*_ is the *p* value of binding site *i* of the top *n* outputs by the PEPSITE algorithm. The total score *S* ^*total*^ for a peptide was calculated using the following equation:

2 $$ S^{total} = \frac{1}{m}\sum\limits_{k = 1}^{m} {(S_{k}^{sub} (1 + \log \frac{{S_{k}^{sub} }}{{S^{core} }})} ), $$

where *m* is the number of all sub-sequences; *S*_*k*_^*sub*^ and *S* ^*core*^ are the binding scores for sub-sequence *k* and that of the core “RKRKKSR” motif calculated using Eq. (1), respectively.

#### Evaluation of Polypeptides’ Binding Affinity for VEGFR Using In Vitro Receptor Binding Assays

A549 cells were cultured in 24-well culture plates at 3 × 10^4^ cells/well in duplicate for each treatment. Cultures were maintained at 37 °C in a 5 % CO_2_ incubator. After 24 h, the culture supernatant was discarded, and cells were washed in PBS, followed by the addition of serum-free culture medium (Hyclone, Logan, UT) before performing an in vitro competitive-binding assay. To each well, VEGF_125–136_ or optimized peptides were added at the following concentrations: 0, 1.3, 6.5, 32.5, 65, 650, and 6,500 nmol/L. After a 30-min incubation period at 4 °C, 1.85 kBq ^125^I-VEGF_165_ (PerkinElmer,Boston, MA) at a final concentration of 0.37 μg/L was added to each well, and the plates were further incubated for another 2 h at 4 °C. The culture supernatant was discarded, and the cells were washed twice with pre-cooled (4 °C) PBS containing 0.1 % BSA to remove unbound ^125^I-VEGF_165_. The cells were then trypsin digested and quickly filtered through glass fiber filter paper using a multi-channel microfluidic device. Radioactive counts (cpm) on the filter paper were determined using a γ-counter (Chongqing Optical & Electrical Instrument). Peptide competition binding curves were plotted, and IC_50_ values for each peptide were calculated using SPSS13.0 statistics software.

#### Identification of Peptide-Binding Sites on A549 Cells Using Fluorescence Microscopy

The highest affinity peptides, NO.15 (QKRKRKKSRKKH) and NO.17 (RKRKRKKSRYIVLS), were conjugated with a fluorochrome, and fluorescence microscopy was used to observe binding to A549 cells as follows. A549 cells were plated on coverslips and grown overnight. The cells were then washed three times with PBS and fixed with 4 % paraformaldehyde for 15 min at room temperature. The cells were incubated at 37 °C for 30 min with 2 % BSA to block nonspecific binding, and subsequently washed three times with PBS. Rhodamine-conjugated peptides (45 µmol/L) were added and incubated with the cells for 2 h at 4 °C. After three washes with PBS, fluorescence microscopy (maximum absorption wavelength of 552 nm) was used to observe cellular staining. Unconjugated rhodamine was used as staining control.

## Results

### Analysis of Molecular Docking and Surface Functional Group Interactions

#### Screening for Polypeptides with High Affinity for VEGFR Using Molecular Docking Analysis

Simulated VEGF_125–136_ was docked semi-flexibly with VEGFR using AutoDock-Vina. The free binding energy was calculated five times, and the lowest binding energy score (–5.0 kcal/mol) was considered as the affinity score for the interaction between VEGF_125–136_ and VEGFR. In all, we identified 17 polypeptides with binding energy lower than −5.2 kcal/mol using a hill-climbing algorithm (Table 1).

**Table 1:** Peptide sequences with VEGFR-binding energy lower than −5.2 kcal/mol (according to molecular docking)

Number	Sequence	Binding energy (kcal/mol)
1	QFRKRKKSRYPK	−6.2
2	QKRKRKKSRYPK	−6.0
3	QFRKRKKSRYPS	−5.9
4	QKRKRKKSRYKY	−5.8
5	QFRKRKKSRWKS	−5.6
6	QKRKRKKSRYKK	−5.5
7	QFRKRKKSRYKS	−5.4
8	KFRKRKKSRYKS	−5.4
9	FFRKRKKSRYKS	−5.3
10	QFRKRKKSRYKH	−5.3
11	HFRKRKKSRYKS	−5.3
12	QFRKRKKSRYLS	−5.3
13	QFRKRKKSRYKF	−5.2
14	QFRKRKKSRYKP	−5.2
15	QFRKRKKSRYKQ	−5.2
16	DKRKRKKSRYKS	−5.2
17	QKRKRKKSRYWS	−5.2
VEGF_125–136_	QKRKRKKSRYKS	−5.0

#### Screening of Polypeptides with Higher Affinity for VEGFR Using Surface Group Interaction Analysis

We predicted the binding region of “RKRKKSR” motif to be on the surface of extracellular domains of VEGFR using PEPSITE (Fig. 3a, b). The predicted sites are consistent with experimental results reported in the literature, and are well matched with the residue positions in the structure of the peptide. This provided important information with respect to the binding location of the core motif on the receptor. This was helpful in peptide design because the segment containing the core motif was absent in the crystal complex of VEGF and VEGFR.

We retained the core sequence (RKRKKSR) of VEGF_125–136_ while gradually adding hydrophilic basic amino acids (R, K, and H) at the N- and C-terminus of the core sequence; alternatively, we replaced specific amino acids in the VEGF_125–136_ sequence with hydrophilic basic amino acids. Furthermore, we added a certain number of basic amino acids to the core peptide, keeping the maximum length at 14 residues. Strong hydrophobic amino acids (I, L, V, and F) were added either at the N- or C-terminus of the sequence. A novel scoring scheme, based on the PEPSITE output, was adopted to produce the peptide-binding score on the receptor protein surface (Eqs. 1 and 2), with higher scores indicating stronger binding (Tables 2 and 3).

**Table 2:** Effect of adding basic groups to the VEGF_125–136_ sequence on PEPSITE score

Number	Sequence	Score calculated in modified PEPSITE (integrated score *S*)
1	QKRKRKKSRKKH	36.8
2	RKRKRKKSRKKH	36.8
3	KKRKRKKSRKRK	36.2
4	QKRKRKKSRYRK	35.6
5	QHRKRKKSRKRH	34.9
6	RKRKRKKSRYKK	34.7
7	QKRKRKKSRYKK	30.8
8	QKRKRKKSRRKK	29.5
9	QKRKRKKSRKK	28.8
10	QKRKRKKSRHKK	28.4
VEGF_125–126_	QKRKRKKSRYKS	27.5

**Table 3:** Effect of adding hydrophobic groups to the VEGF_125–136_ sequence on PEPSITE score

Number	Sequence	Score calculated in modified PEPSITE (integrated score *S*)
1	QHKRKRKKSRIVL	30.3
2	KFRKRKKSRYIV	29.6
3	RKRKRKKSRYIVLS	28.8
4	IVFKRKRKKSRYLS	28.6
5	RKRKRKKSRKIVL	27.1
6	ILLVRKRKSRYKK	25.9
7	IVVRKRKSRYKH	25.4
8	IVVRKRKSRYRK	25.1
9	ILIVRKRKSRYKK	24.3
10	RKRKKSRKKHIL	24.1
VEGF_125–136_	QKRKRKKSRYKS	23.7

Thus, we selected 20 peptides with predicted outcomes better than VEGF_125–136_ as candidate sequences (Table 4).

**Table 4:** Twenty peptides with theoretical VEGFR-binding affinities exceeding that of VEGF_125–136_ and IC_50_ values from competitive VEGFR binding with ^125^I-VEGF

Number	Sequence	IC50 (nmol/L)
Control	VEGF_125–136_	464 ± 12
15	QKRKRKKSRKKH	80 ± 7
5	QFRKRKKSRWKS	92 ± 9
17	RKRKRKKSRYIVLS	185 ± 14
10	FFRKRKKSRYKS	188 ± 25
11	QFRKRKKSRYKH	252 ± 16
1	QFRKRKKSRYPK	273 ± 19
9	KFRKRKKSRYIV	273 ± 33
4	QKRKRKKSRYKY	280 ± 13
18	RKRKRKKSRKKH	283 ± 9
19	KKRKRKKSRKRK	315 ± 11
14	QFRKRKKSRYKF	323 ± 17
20	QKRKRKKSRYRK	405 ± 20
16	QHKRKRKKSRIVL	424 ± 37
8	QKRKRKKSRYPLS	542 ± 16
6	QKRKRKKSRYKK	571 ± 40
2	QKRKRKKSRYPK	602 ± 19
12	IVFKRKRKKSRYLS	637 ± 25
7	QFRKRKKSRYKS	665 ± 38
13	QFRKRKKSRYLS	694 ± 29
3	QFRKRKKSRYPS	4206 ± 76

### Evaluation of Polypeptides’ Binding Affinity for VEGFR

**Fig. 4:**
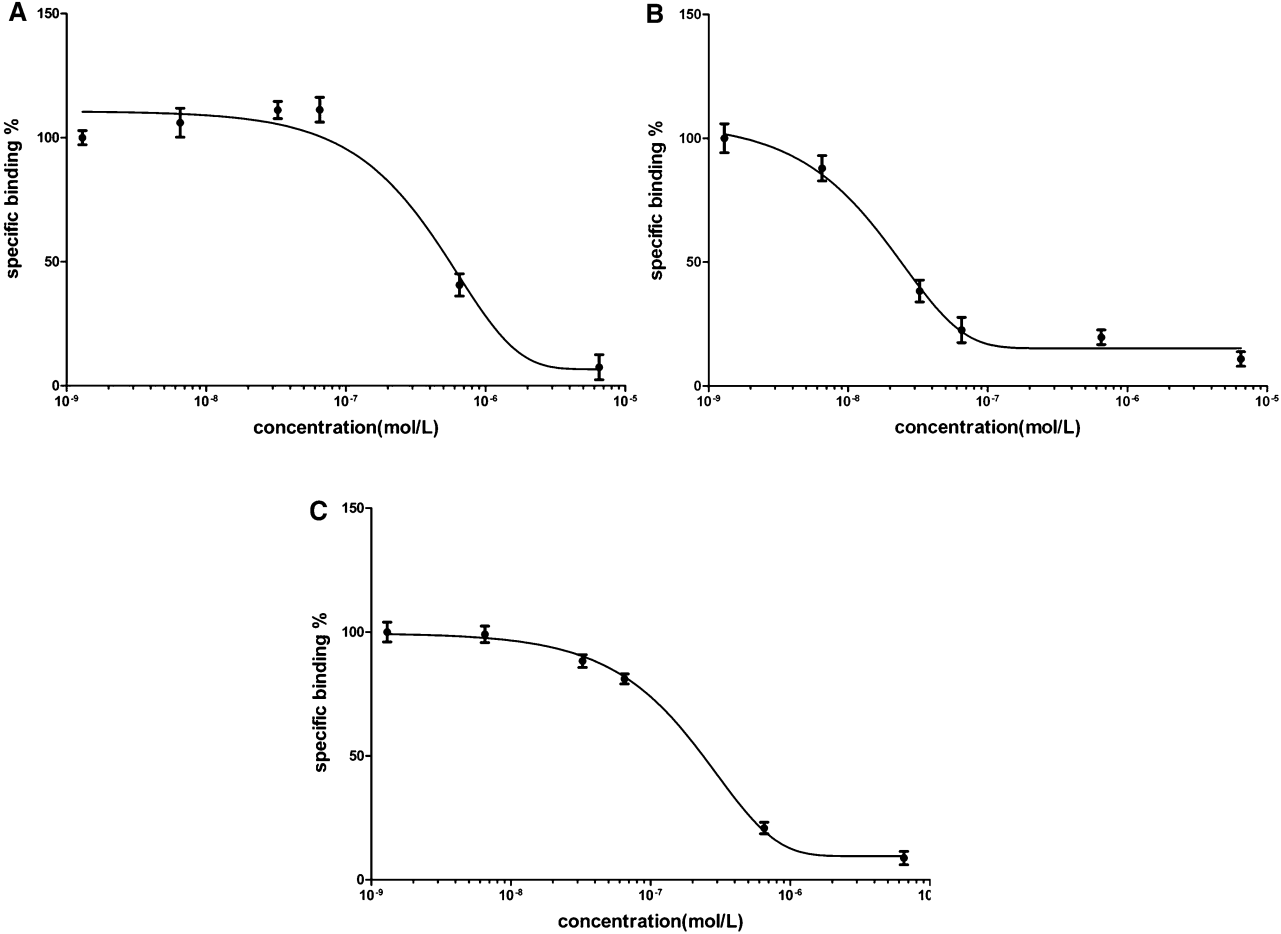
VEGFR peptide binding inhibition curves. Analysis of ^125^I-VEGF binding in the presence of VEGF_125–136_ (**a**), QKRKRKKSRKKH (**b**), or RKRKRKKSRYIVLS (**c**) using A549 cells resulted in IC_50_ values of 464, 80, 185 nmol/L, respectively. The IC_50_ value of each peptide was calculated using the SPSS13.0 statistics software package

All 20 selected peptides produced potent dose-dependent inhibition of ^125^I-VEGF binding to VEGFR, which suggests that all 20 peptides bound specifically to VEGFR. Thirteen polypeptides exhibited IC_50_ values lower than VEGF_125–136_ (464 nmol/L, Fig. 4a), with the IC_50_ values of the remaining polypeptides exceeded this value. The IC_50_ values of QKRKRKKSRKKH and RKRKRKKSRYIVLS were significantly lower than that of VEGF_125–136_, at 80 nmol/L (Fig. 4b) and 185 nmol/L (Fig. 4c), respectively (Table 4). This corresponds to 6- and 2.5-fold increases in affinity for QKRKRKKSRKKH and RKRKRKKSRYIVLS, respectively. Blast searches of the NCBI database revealed that both peptides were novel.

### Evaluation of Peptides Binding in A549 Cells Using Fluorescence Microscopy

**Fig. 5:**
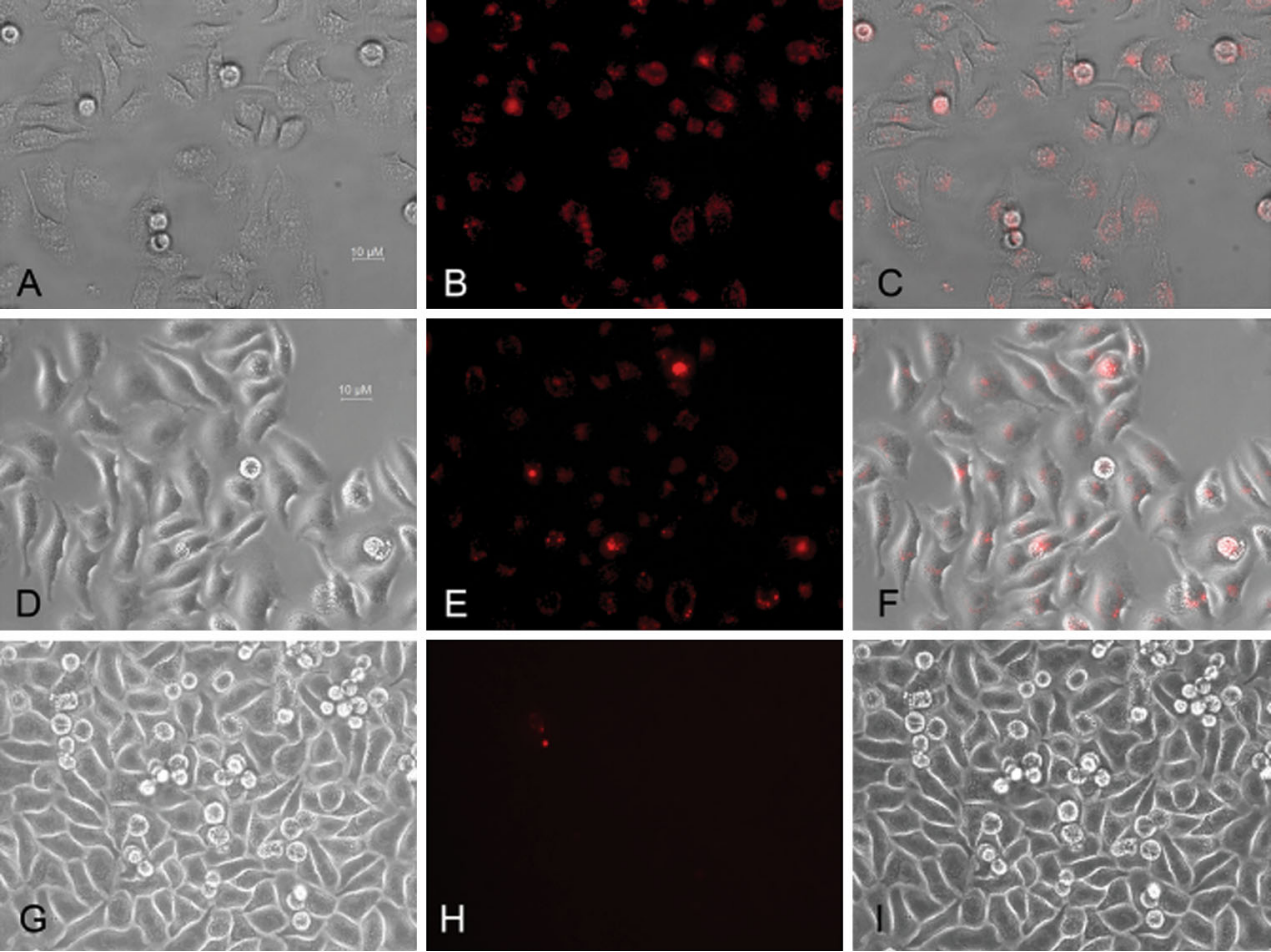
Binding of rhodamine-conjugated peptides to A549 cells in vitro Rhodamine-labeled peptides were incubated with A549 cells for 6 h after which bright field (**a** QKRKRKKSRKKH, **d** RKRKRKKSRYIVLS, **g** rhodamine),sssss and red-fluorescent (**b** QKRKRKKSRKKH, **e** RKRKRKKSRYIVLS, **h** unconjugated-rhodamine) images were obtained; merged images (**c** QKRKRKKSRKKH, **f** RKRKRKKSRYIVLS, **i** unconjugated-rhodamine) were generated at a 400× magnification. Unconjugated rhodamine was used as the negative control

The binding of rhodamine-conjugated peptides is shown in Fig. 5. After incubation with the A549 cells for 6 h, specific labeling was observed using fluorescence microscopy. In contrast, incubation with unconjugated rhodamine did not result significant levels of fluorescent staining of A549 cells. Analysis of the merged bright field and fluorescent images demonstrated that specific binding of peptides NO.15 (QKRKRKKSRKKH) and NO.17 (RKRKRKKSRYIVLS) is observed in the plasma membrane and perinuclear cytoplasm of A549 cells.

## Discussion

Angiogenesis is an essential process involved in normal tissue development as well as for carcinogenesis of solid tumors and metastasis. VEGF plays a critical role in tumor angiogenesis and blocking VEGF/VEGFR signaling significantly inhibits tumor growth and metastasis [13, 23, 24]. Thus, targeting VEGF/VEGFR with molecular probes holds great promise for use in radionuclide imaging or therapeutic applications.

An important criterion for effective polypeptide targeting is the affinity between a polypeptide and its targeted receptor, where higher affinity results in increased specificity and stronger targeting capability. Higher affinity binding produces decreased dissociation of the peptide from its receptor, resulting in higher tissue peptide concentrations with longer residency times. Our previous work showed that VEGF_125–136_ exhibits good tumor-targeting properties [20]. In this study, we aimed to identify peptides with higher VEGFR-binding affinity with enhanced tumor-targeting effects that could be used in molecular imaging and to substantially inhibit the growth of tumor cells or tumor vasculature endothelial cells.

Bioinformatic approaches enable the prediction and in silico selection of large numbers of candidate sequences at a relatively low cost and higher efficiency than through conventional biological experimentation. Molecular design methods employing the structure and interaction of bio-macromolecules are gradually gaining ground in the study of polypeptides and drug design. Exploiting molecular docking and surface group interaction analyses, we designed 20 polypeptides with theoretically higher VEGFR-binding affinity than VEGF_125–136_. As verified using biological methods, polypeptides selected using molecular docking had lower binding affinities than those identified by surface group interaction analysis; i.e., the predictive value of the former method is less optimal than the latter. This is likely to be due to the instability of polypeptide structures, as well as the complexity and vast number of calculations required for molecular docking analysis. In future studies, we plan to modify the molecular docking approach employed here and to perform a thorough molecular dynamics simulation to further improve the validity of molecular docking data in predicting and assessing the interaction between polypeptides and their receptors.

Using our bioinformatic strategy, we ultimately obtained 20 peptide candidates. Since the binding characteristics of these peptides were calculated in silico, biological experiments were required to verify the accuracy of the predictions and to positively identify the optimal peptide(s). Thus, competitive-binding assays were employed to determine the actual IC_50_ values with A549 cells. A549 cells are a human lung adenocarcinoma cell line that expresses high-levels of VEGFRs. These experiments used ^125^I-VEGF_165_ as the standard protein, which has the greatest binding affinity for VEGFR. The observation of competitive binding with VEGF_165_ indicates that the peptide candidates had the capacity to bind the VEGFR. Using this method, we found that the computer predictions were not completely consistent with the biological experimental results. Peptides QKRKRKKSRKKH (NO.15) and RKRKRKKSRYIVLS (NO.17) exhibited the lowest IC_50_ values in competitive binding to VRGFR, which indicates that these two peptides had the highest binding affinities.

Visualization of the cellular localization of peptide-binding sites was performed using fluorochrome-conjugated peptide staining of A549 cells. A549 cells were incubated with rhodamine-conjugated peptides and observed with fluorescence microscopy. These experiments indicated that labeling was observed mainly on the plasma membrane and in the perinuclear cytoplasm of A549 cells. These results are consistent with peptides targeting cell-surface receptors as well as undergoing intracellular transport through receptor-mediated internalization. Conversely, no significant fluorescent staining was observed in the control group, which used unconjugated rhodamine. This finding indicates that the peptides designed herein specifically bound VEGF receptors on A549 cells.

The polypeptides QKRKRKKSRKKH and RKRKRKKSRYIVLS exhibited ≈6- and 2.5-fold higher VEGFR-binding affinity than VEGF_125–136_, respectively. This finding suggests that these two peptides could be used as novel therapeutic agents or as molecular probes for tumor-targeted radionuclide imaging.

## Conclusion

Bioinformatic methods were successfully employed to identify two potential tumor-targeting small-molecule peptides with higher affinity for VEGFR than VEGF_125–136_. This study establishes the experimental basis for further research into VEGFR targeting of tumors for molecular imaging and therapy.
